# Case report: Bilateral double beta peak activity is influenced by stimulation, levodopa concentrations, and motor tasks, in a Parkinson’s disease patient on chronic deep brain stimulation

**DOI:** 10.3389/fneur.2023.1163811

**Published:** 2023-05-18

**Authors:** Giulia Giannini, Luca Baldelli, Gaetano Leogrande, Ilaria Cani, Paolo Mantovani, Giovanna Lopane, Pietro Cortelli, Giovanna Calandra-Buonaura, Alfredo Conti

**Affiliations:** ^1^UOC Clinica Neurologica Rete Metropolitana NEUROMET, IRCCS Istituto delle Scienze Neurologiche di Bologna, Bologna, Italy; ^2^Department of Biomedical and NeuroMotor Sciences (DiBiNeM), Alma Mater Studiorum - University of Bologna, Bologna, Italy; ^3^Medtronic EMEA Corporate Technology and Innovation, Maastricht, Netherlands; ^4^Unit of Neurosurgery, IRCCS Istituto delle Scienze Neurologiche di Bologna, Bologna, Italy; ^5^Unit of Rehabilitation Medicine, IRCCS Istituto delle Scienze Neurologiche di Bologna, Bologna, Italy

**Keywords:** Parkinson’s disease, deep brain stimulation, local field potentials, beta band frequency, case report

## Abstract

**Introduction:**

Subthalamic (STN) local field potentials (LFPs) in the beta band are considered potential biomarkers for closed-loop deep brain stimulation (DBS) in Parkinson’s disease (PD). The beta band is further dissected into low-and high-frequency components with somewhat different functions, although their concomitance and association in the single patient is far to be defined. We present a 56-year-old male PD patient undergoing DBS showing a double-beta peak activity on both sides. The aim of the study was to investigate how low-and high-beta peaks were influenced by plasma levodopa (L-dopa) levels, stimulation, and motor performances.

**Methods:**

A systematic evaluation of raw LFPs, plasma L-dopa levels, and motor tasks was performed in the following four conditions: OFF medications/ON stimulation, OFF medications/OFF stimulation, ON medications/OFF stimulation, and ON medications/ON stimulation.

**Results:**

The analysis of the LFP spectra suggests the following results: (1) the high-beta peak was suppressed by stimulation, while the low-beta peak showed a partial and not consistent response to stimulation; (2) the high-beta peak is also influenced by plasma L-dopa concentration, showing a progressive amplitude increment concordant with plasma L-dopa levels, while the low-beta peak shows a different behavir; and (3) motor performances seem to impact beta peaks behavior.

**Conclusion:**

This single exploratory case study illustrates a complex behavior of low-and high-beta peaks in a PD patient, in response to stimulation, L-dopa plasma levels, and motor performances. Our results suggest the importance to investigate patient-specific individual LFP patterns in view of upcoming closed-loop stimulation.

## Introduction

Parkinson’s disease (PD) is a movement disorder characterized by nigrostriatal dopamine depletion. The emergence of stereotyped patterns of oscillations within cortico-basal-ganglia circuits in these patients has gained the spotlight as a solid way of exploring PD pathophysiology (1). Deep brain stimulation (DBS) offers a unique opportunity to record this pathological electrophysiological activity by means of local field potentials (LFPs) that are coming from the discharges of a cluster of neurons surrounding the depth electrode usually implanted in the subthalamic nucleus (STN) (2). This is of particular relevance with recently developed implantable pulse generators (IPG; Medtronic Percept™ PC, Medtronic PLC, United States) that allow chronic simultaneous recording and electrical stimulation of basal ganglia (3, 4).

The sensing capabilities of these new implantable devices have allowed a better understanding of disease-related brain activity patterns and their modulation in response to therapies, bringing the implementation of adaptive stimulation therapies closer to clinical practice.

These sensing capabilities include the collection, in clinic, of differential raw time domain LFP, sampled at 250 Hz, from a predefined contact pair per hemisphere, and the visualization in real time of the magnitude of a LFP’s predefine frequency band (Brainsense™ Streaming). Moreover, both inside and outside the clinic, when the user marks an event via the patient programmer, the neurostimulator stores the LFP’s spectrum from a predefined contact pair per hemisphere (Brainsense™ Events). This information can be retrieved in a report in json format for offline analysis. Both these recording modes are possible in ON or OFF stimulation (5).

The most studied and debated STN LFP oscillations are in the beta range (13–35 Hz), as experiments in PD patients have demonstrated abnormally sustained and synchronized oscillations in this frequency span (6, 7). These oscillations are thought to be mechanistically involved in symptom manifestation by distorting the communication between brain areas needed for the initiation of voluntary movement (2, 8). The amplitude of beta oscillations correlates with the severity of akinetic/rigid symptoms (9, 10), and importantly, their reduction following DBS positively correlates with motor improvement (11, 12). Recent studies have further dissected the band into two separate frequency components with somewhat different functions. Low-beta peaks represent the frequency band of 13–20 Hz, and high-beta peaks represent the frequency band of 21–35 Hz. In general, low beta is regarded as a purely pathological oscillation (10), while high beta seems to retain a physiological role (13), although, their concomitance and their association in a single patient need to be defined.

We present a 56-year-old male PD patient undergoing DBS showing a double beta peak activity on both sides, in the low-and high-beta range.

## Case report

The patient showed an akinetic-rigid phenotype and a disease duration of 7 years. Two years before surgery, he developed severe motor fluctuations. At the time of the evaluation, he presented early morning OFF, nocturnal akinesia, disabling wearing OFF, and peak-dose dyskinesia. He assumed levodopa (L-dopa) every 2 h during the day with a levodopa equivalent daily dose (LEDD) (14) of 2,247 mg.

The preoperative evaluation showed a levodopa-responsive freezing of gait and a good L-dopa response (48%) calculated, using the Movement Disorders Society—Unified Parkinson’s Disease Rating Scale (MDS-UPDRS) (15), as follows: [(preoperative “OFF” medication MDS-UPDRS III score − preoperative “ON” medication MDS-UPDRS III score)/preoperative “OFF” medications MDS-UPDRS III score] × 100.

A thorough neuropsychological evaluation, a psychiatric interview, and a 3-Tesla MRI showed no contraindications. He underwent DBS surgery in September 2020. Electrode leads (Medtronic 3,389™) were implanted into the STN on both sides. Single-cell activity and LFP were recorded during the surgery, and test stimulation was applied to evaluate its effect on rigidity and bradykinesia. In the same session, the pulse generator was implanted (Medtronic Percept™). DBS programming was performed 1 month after surgery in order to avoid the microlesion effect. The initial programming session was performed in the morning after an overnight medication washout to guarantee a stable OFF drug condition in order to prevent medication effects from interfering with DBS effects.

During DBS programming, the amplitude thresholds for clinical benefits and side effects were tested for each electrode contact. For stimulation electrodes #1, #2, #9 #10, raw LFP traces were recorded from the electrodes adjacent to the respective stimulation electrode on each side during rest and streamed wirelessly to a tablet computer.

**Figure 1:**
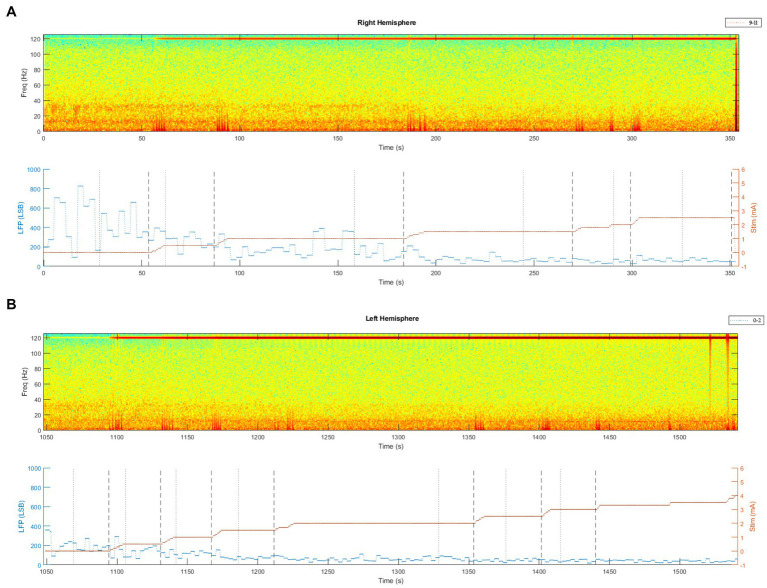
Spectrum of the raw LFP, recorded via the Brainsense™ Streaming, during stimulation titration at the initial DBS programming session. **(A)** Brainsense™ Streaming LFP recordings during stimulation titration in the right STN. In the upper part of the graph: spectrograms extracted from the raw LFP (stimulation at contact #10, sensing at contacts #9 and #11). In the lower part of the graph: LFP plotted over time, together with stimulation titration (mA). **(B)** Brainsense™ Streaming LFP recordings during stimulation titration in the left STN. In the upper part of the graph: spectrograms extracted from the raw LFP (stimulation at contact #1, sensing at contacts #0 and #2). In the lower part of the graph: LFP plotted over time, together with stimulation titration (mA).

The spectra of the LFP, recorded during the initial DBS programming session, showed a double-beta peak activity on both sides in the low- (~17 Hz) and high-beta range (~30 Hz). These beta activities were gradually suppressed by the progressive increase of the stimulation amplitude (Figure 1). In particular, the high-beta peak showed a marked suppression, while the low-beta peak did not consistently decrease. At the end of the monopolar review, to determine the amplitude threshold for clinical benefits and side effects, the following contacts were selected for stimulation and sensing: left STN = stimulation at contact #1, sensing at contacts #0 and #2; right STN = stimulation at contact #10, sensing at contacts #9 and #11. Stimulation was delivered with a frequency of 125 Hz, pulse width of 60 μs, and amplitude of 1.5 mA and 1.2 mA on the left and right STN, respectively. Four events were programmed in the patient controller (dyskinesia, rigidity, freezing, “I’m feeling good”).

At the follow-up visits, the events analysis revealed a prevalence of the peak at ~17 Hz over the peak at higher frequency (~30 Hz) during the events marked for rigidity, and a prevalence of the peak at ~30 Hz, with the almost complete absence of the peak at ~17 Hz during events marked for dyskinesia.

In order to investigate how these beta peaks were influenced by medications, stimulation, and motor state (OFF vs. ON), a systematic evaluation of raw LFP, plasma L-dopa levels, and motor performances was performed in the following four conditions: (1) “OFF” medications and “ON” stimulation (MED-OFF/STIM-ON); (2) “OFF” medications and “OFF” stimulation (MED-OFF/STIM-OFF); (3) “ON” medications and “OFF” stimulation (MED-ON/STIM-OFF); and (4) “ON” medications and “ON” stimulation (MED-ON/STIM-ON). In addition to these experiments, we tested the effect of motor performances on LFP in the same patient.

## Methods

### Study protocol

This evaluation was performed 6 months after surgery, after a 12-h washout of L-dopa and any concomitant antiparkinsonian drugs. In addition to dopaminergic drugs, the patient took clonazepam 0.5 mg before bedtime. The schematic outline of the study protocol is shown in Supplementary Figure S1.

Blood venous samples (2 mL) for the measurement of plasma L-dopa concentrations were drawn by an indwelling catheter in medications OFF conditions and at 15-min intervals for the first 90 min after dosing. Blood specimens were collected and processed for plasma L-dopa analysis, as previously reported (16).

In each condition, motor performances were evaluated by the following tests:

1. Alternate index finger tapping test: This test objectively measures the number of times the patient can alternately tap two buttons 20 cm apart in 60 s with the most affected hand (the right hand for this patient), using a computerized touch screen system (17)
2. Timed up and go test (TUGT) (18)
3. MDS-UPDRS III score (15).

The percentage of postoperative motor improvement was calculated as follows: [(preoperative “OFF” medications MDS-UPDRS III score − postoperative “OFF” medications and “ON” stimulation MDS-UPDRS III score)/preoperative “OFF” medications MDS-UPDRS III score] × 100.

The preoperative and postoperative LEDD were calculated (14), along with LEDD change calculated as follows: (preoperative LEDD – postoperative LEDD)/preoperative LEDD.

Finally, the preoperative and postoperative MDS-UPDRS I, II, and IV scores (15) and the Hoehn and Yahr scale (H&Y) score (19) were measured.

### LFP analysis

For each condition, the raw LFPs (one channel from each electrode lead) were sampled at 250 Hz and streamed wirelessly to a tablet computer for about 5-min in resting-state condition and during the tasks described above. The power spectral density estimate (PSD, obtained using the Welch’s method with 1-s Hamming’s window, overlap of 60%, and Fast Fourier Transform (FFT) size of 250) and the spectrogram were computed from the resting state LFP time domain signals recorded via the Brainsense™ Streaming feature to compare the signals in each condition. For consistency, only the last 3 min of the recordings in resting state LFP in each condition was used for this analysis. Moreover, Brainsense™ events were recorded in “OFF” medications and “ON” stimulation, in “OFF” medications and “OFF” stimulation, at 15-min intervals after dosing (simultaneously with blood sample collection) for the first 60 min, in “ON” medications and “OFF” stimulation (75 min after dosing), and in “ON” medications and “ON” stimulation. For each condition, power spectra were normalized to the percentage of total power of 5–45 Hz and 55–95 Hz and are further expressed as a percent of total power. The 0–5 Hz and 45–55 Hz ranges were omitted to avoid contamination by movement artifact and main noise, respectively (10).

Peaks were defined as the maximum in normalized spectral power of the raw LFPs or of the recorded events in bands (low beta: 10–16 Hz and high beta: 26–34 Hz) defined arbitrarily based on the peaks identified in the baseline condition OFF medications and OFF stimulation or, in absence of a maximum in such bands, as the value of the PSD at the frequency of the peak in the MED-OFF/STIM-OFF condition. The amplitude of the peaks in the low-beta and high-beta frequency bands were plotted, together with the L-dopa concentration, over time.

Finally, the spectra of the raw LFPs were compared for the three different motor tasks in the same condition. In particular, the entire time frame of the alternate index finger tapping test and TUGT was included in the LFP analysis; regarding MDS-UPDRS part III, the time period where bradykinesia, posture, and gait items were performed was included, while facial expression, speech, rigidity, and tremor items were excluded from LFP evaluation.

## Results

**Figure 2:**
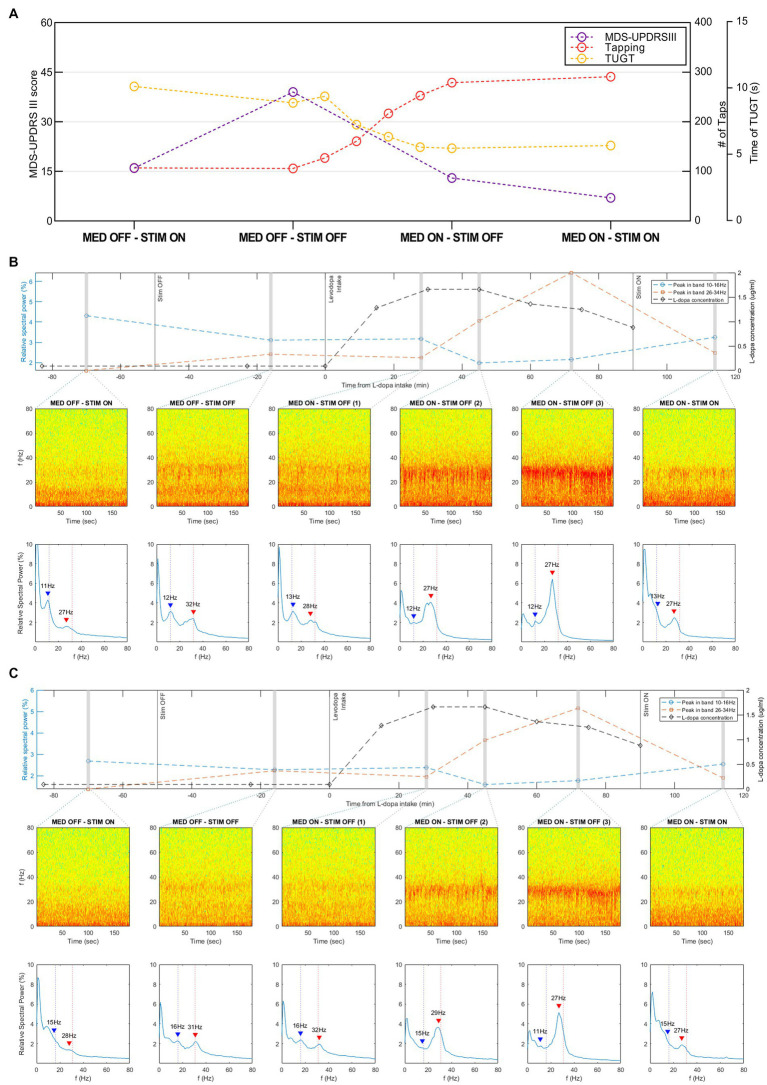
Motor outcomes and recordings in resting state, via the Brainsense™ Streaming, of the STN-LFP in different conditions. **(A)** Motor outcomes (MDS-UPDRS III, alternate index finger tapping test, and TUGT scores) in the four conditions (MED-OFF/STIM-ON, MED-OFF/STIM-OFF, MED-ON/STIM-OFF, and MED-ON/STIM-ON). **(B)** Recordings in the resting state of the LFP in the right STN recorded via the Brainsense™ Streaming in different conditions. In the mid part of the graph: Spectrograms extracted from the raw LFP. In the lower part of the graph: the relative spectral power of the raw LFP in the frequency range 0–80 Hz. Blue and red arrows highlight the identified peaks in the low−/high-beta frequency band, respectively. In the upper part of the graph: relative spectral power of the peaks in the low-beta and high-beta frequency bands plotted over time, together with the L-dopa concentration. **(C)** Recordings in the resting state of the LFP in the left STN recorded via the Brainsense™ Streaming in different conditions. In the mid part of the graph: Spectrograms extracted from the raw LFP. In the lower part of the graph: the relative spectral power of the raw LFP in the frequency range 0–80 Hz. Blue and red arrows highlight the identified peaks in the low−/high-beta frequency band, respectively. In the upper part of the graph: relative spectral power of the peaks in the low-beta and high-beta frequency bands plotted over time, together with the L-dopa concentration. L-dopa, levodopa; MDS-UPDRS, Movement Disorder Society-Unified Parkinson’s Disease Rating Scale part III, motor subsection; MED-OFF, OFF medications; MED-ON, ON medications; STIM-OFF, OFF stimulation; STIM-ON, ON stimulation; TUGT, timed up and go test.

Stimulation led to a remarkable improvement in the patient’s symptoms and motor fluctuations. MDS-UPDRS III, alternate index finger tapping test, and TUGT scores, gathered in the four conditions, are presented in Figure 2A.

The postoperative motor improvement, calculated as indicated in the method section, resulted of 60%. The I and II sections of the MDS-UPDRS changed from 10 to 5 and from 8 to 3, respectively. Motor complications, measured by the MDS-UPDRS part IV, improved changing from a preoperative score of 9 to a postoperative score of 3. The H&Y score remained unchanged (score 2). The LEDD change, calculated as indicated in the method section, resulted of 0.54 (pre-operative = 2247 mg, post-operative 1042 mg).

The spectra of the LFPs showed a different double-peaked beta activity trend during the four conditions.

On the right hemisphere, the high-beta peak’s relative spectral power increased after stimulation was turned OFF (amplitude from 1.6 to 2.4%). After L-dopa intake the high-beta peak progressively increased from 25 to 70 min after dosing, reaching a relative spectral power amplitude of 6.4%, and subsequently decreased to 2.5% (Figure 2B). The analysis of events recording showed that this decrease started 60 min after dosing and was independent of the stimulation (Supplementary Figure S2A). The high-beta peak values showed a similar, although delayed, trend with L-dopa concentration, which started to increase at 15-min after dosing, reached a plateau between 30 and 45 min, and subsequently decreased.

The analysis of the high-beta activity on the left hemisphere derived both from streaming and events recordings showed a similar behavior (Figure 2C; Supplementary Figure S2B). The relative spectral power of this peak was 1.4% in MED-OFF STIM-ON condition, increased to 2.2% when stimulation was turned OFF, progressively increased after L-dopa intake reaching a value of 5.2%, and subsequently decreased to 1.9%. Taking the high-beta peak at MED-OFF STIM-OFF condition as a reference, we observed, on each side, a frequency shift from 31 to 32 Hz to 27–28 Hz after both L-dopa intake and stimulation switch-ON (Figures 2B,C).

The lower beta peak showed a different behavior in response to L-dopa concentrations progressively decreasing in amplitude after dosing (from 3.1 to 2.0% on the right STN and from 2.3 to 1.6% on the left STN) (Figures 2B,C). Notably, the low-beta peak’s relative spectral power did not undergo a relevant change when stimulation was turned OFF from 1.2 mA of current, resulting in a slight decrease. During the analysis of events recording, on both sides, this peak showed a progressive decrease after dosing and a subsequent suppression when stimulation was turned ON (Supplementary Figures S2A,B).

**Figure 3:**
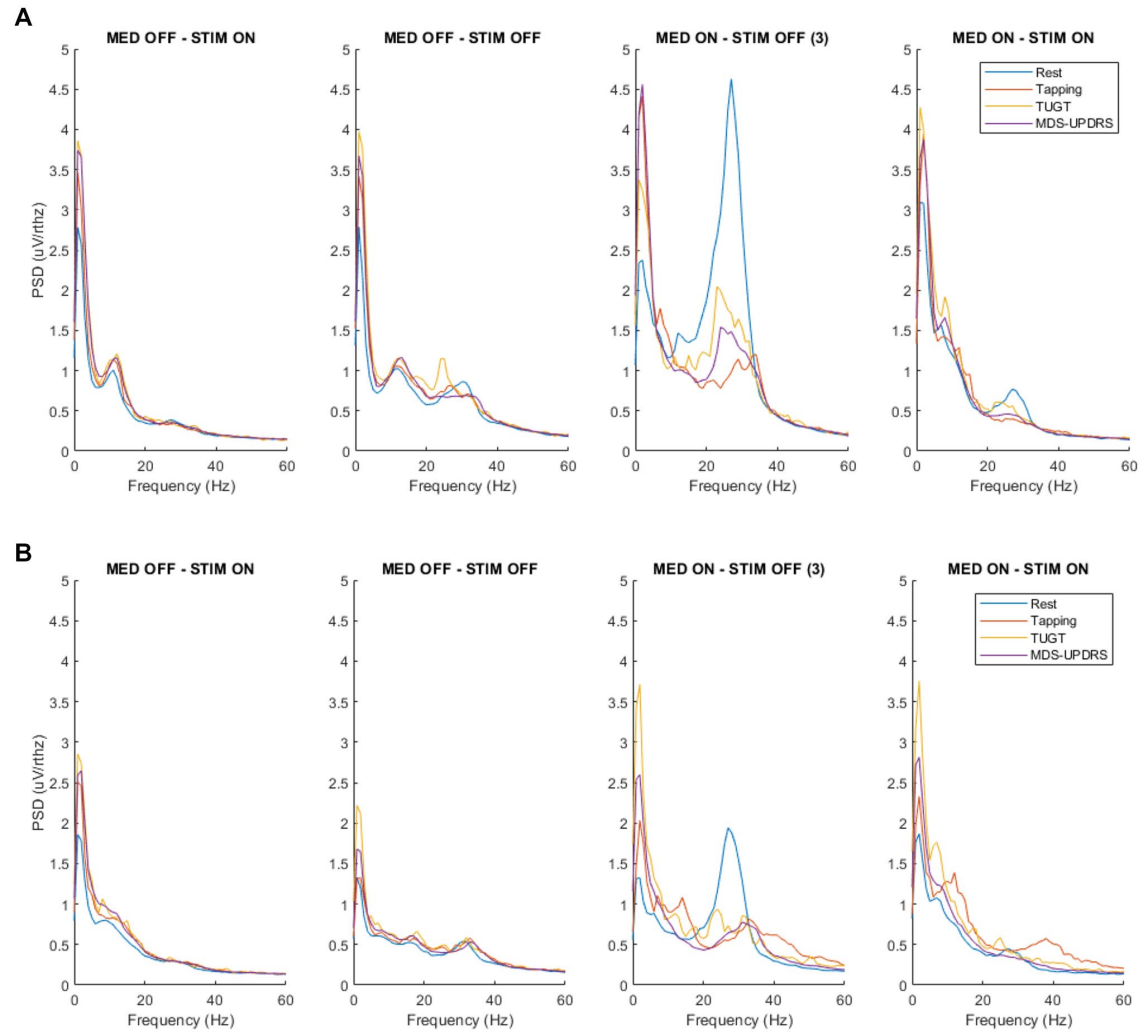
Spectrum of the raw LFP compared for different tasks in the four conditions, recorded via the Brainsense™ Streaming. **(A)** Spectrum of the raw LFP of the right side. **(B)** Spectrum of the raw LFP of the left side. MDS-UPDRS, Movement Disorder Society-Unified Parkinson’s Disease Rating Scale part III, motor subsection; MED-OFF, OFF medications; MED-ON, ON medications; rest, resting state; STIM-OFF, OFF stimulation; STIM-ON, ON stimulation; tapping, alternate index finger tapping test; TUGT, timed up and go test.

Considering the effect of motor tasks on LFP in the same patient, gait, tapping, and MDS-UPDRS evaluation had a suppressive effect on the high-beta peak (Figure 3). Under stimulation, motor performances had an additional suppressive effect (motor performances suppressed high-beta peak almost completely when the stimulation was ON). Motor tasks seemed to play a different role on the low-beta peak, thus slightly increasing the amplitude. This was mainly detectable in the MED-OFF condition, when the low-beta peak was more prevalent over the peak at a higher frequency.

## Discussion

This single exploratory case study illustrates a complex behavior of low-and high-beta peaks in a PD patient, in response to stimulation, L-dopa plasma levels, and motor performances.

Regarding this patient, the analysis of the LFP spectra suggests the following results: (1) the high-beta peak was clearly suppressed by stimulation, while the low-beta peak showed a partial and not consistent response to stimulation; (2) the high-beta peak is also influenced by plasma L-dopa concentration, showing a progressive amplitude increment concordant (though delayed) with plasma L-dopa levels, while the low-beta peak shows a progressive decrease after dosing; (3) motor performances seem to impact beta peaks behavior.

These results illustrate the prospects of using novel neurostimulation devices that allow continuous recording of LFP in addition to delivering DBS to the respective target structure.

As compared with previous studies recording beta peaks in PD patients in the postoperative window, our results showed the relationship between beta peaks activity and standardized L-dopa plasma levels measurements. A better characterization of beta activity could deepen the knowledge of beta peaks’ behavior in response to DBS, medications, and motor pattern, thus, having important implications for LFP-controlled closed-loop stimulation. If raised L-dopa plasma concentrations result in an increased peak, as seen in our recordings, this may affect the usefulness of LFP as a feedback signal for closed-loop stimulation. For this reason, while the high-beta peak showed a marked response to stimulation, its relevant increase concomitant with higher L-dopa plasma concentrations might be assumed as a discordant and tricky feature for a possible adaptive stimulation.

Previous studies (20–23) showed that a clinically effective L-dopa dose decreases beta oscillations, particularly those in the low-beta (13–20 Hz) frequency range, in accordance with our results. One study investigated changes, induced by L-dopa and DBS on STN beta LFP oscillations, in nine patients with PD under four experimental conditions: MED-OFF/STIM-OFF, MED-OFF/STIM-ON, MED-ON/STIM-ON, and MED-ON/STIM-OFF (24). In this study, each nucleus was characterized to detect a peak in the beta range, and a significant beta peak on a low-beta band (central frequency from 13.6 to 16.6 Hz) was found in six out of nine patients. The analysis of STN LFP oscillations showed that L-dopa abolished beta STN oscillations in all the patients, while DBS significantly decreased the beta oscillation only in five of the nine patients studied. Moreover, while L-dopa completely suppressed beta oscillations, DBS merely decreased them (24).

However, literature on high-beta band behavior in response to L-dopa is lacking and inconsistent. A pioneer study investigated the pharmacological modulation of STN activity, recording LFP 2–3 days after the electrode implantation on 13 PD patients (23). After acute L-dopa administration, beta oscillations significantly decreased in the low-beta range (13–20 Hz; log power change = 0.70 ± 0.51; *p* = 0.0007) and though less consistently in the high-beta range (20–30 Hz; log power change = 0.25 ± 0.40; *p* = 0.0479). A low-amplitude but well-tuned high-beta rhythm was still present after L-dopa, both in the average spectrum and in the superimposed spectra of individual nuclei (23). In a recent study, neural activity was recorded in the cortex and basal ganglia of healthy non-human primates while acutely and chronically up-and down-modulating dopamine levels (25). In the same study, changes in beta oscillations were assessed in four PD patients following acute and chronic changes in dopamine tone. The analysis of LFP beta oscillations properties in healthy non-human primates and PD patients revealed that the amplitude of the peak and area under curve, in beta range, were inconsistent after acute treatment. On the other hand, other beta proprieties such as the frequency of beta oscillations, beta coherence, and phase-amplitude coupling are strongly correlated with acute and chronic changes in dopamine tone (25). These data suggest that further studies, and on a larger PD sample, are necessary to better investigate the high-beta band behavior in response to L-dopa, to evaluate different beta band patterns and their possible correlation with clinical phenotype, identifying which beta proprieties are the most appropriate biomarkers.

Eventually, in our patient, the stimulation suppressed the high-beta peak with partial and non-consistent suppression of the low-beta peak, differing from previous findings on this topic where stimulation suppressed synchronized neuronal activity within the STN, preferentially at low-beta rather than high-beta frequencies (26).

Hence, the results obtained from our patient suggest the importance to investigate patient-specific individual LFP patterns (LFP “fingerprints”) in view of upcoming closed-loop stimulation. Given the high variability among patients, possibly related to the disease stage, phenotype, levodopa-response and levodopa-pharmacokinetic levels, the regulation of sensing and DBS parameters should be designed on the single patient, after a study of his pattern in response to treatment and stimulation. These findings could help to optimize stimulation and to investigate the predictive role of these frequency behaviors on patients’ outcomes.

Further studies on large cohorts, and probably, of multicentric nature, are necessary to further characterize LFP patterns in response to stimulation and medications and to identify specific biomarkers among PD patients’ subgroups and phenotypes, tailoring DBS to the individual patient, preparing for the upcoming closed-loop adaptive stimulation.

Concerning the impact of motor performance on LFP, we compared the spectrum of the raw LFPs for different tasks in the same condition. In our patient, motor tasks had a suppressive effect on the high-beta peak, and, under stimulation, motor performances had an additional suppressive effect. Motor tasks seemed to play a different role on the low-beta peak slightly increasing the amplitude (mainly detectable in MED-OFF condition when the low-beta peak was more prevalent over the peak at higher frequency).

These findings are partially concordant with the results of other previous studies. In the literature, only one study investigated the behavior of double beta peak activity in response to stimulation and physical activity in 1 PD patient undergoing STN-DBS. In this study, the LFP spectra recordings were analyzed during standing (STIM ON and STIM OFF) and walking (STIM ON and STIM OFF). On the left side, gait suppressed beta peaks almost completely when stimulation was OFF, while, on the right side, under stimulation, gait had an additional but incomplete suppressive effect on beta peaks (27).

Other studies focused on LFP modulation by motor performances showing a beta band suppression during movement. One study simultaneously recorded LFP from the STN and/or ipsilateral globus pallidus interna (GPi) or scalp EEG during voluntary movements of a hand-held joystick in six awake patients following neurosurgery for PD. Without medication, the power within the STN and the coherence between the STN and the GPi were dominated by activity with a frequency of <30 Hz. This coupling was attenuated by movement (28). Another study characterized LFP in STN in terms of beta-burst prevalence, amplitude, and length between movement and rest as well as during self-paced as compared to goal-directed motor control. Electrophysiological recordings from externalized DBS-electrodes in nine PD patients showed a marked decrease in beta-burst durations and prevalence during movement as compared to rest as well as shorter and less frequent beta-bursts during cued as compared to self-paced movements (29). One study compared beta power in the STN and GPi during rest and movement in 37 PD patients undergoing DBS. The analysis of recordings obtained shortly after DBS electrodes placement, in the operating room setting, showed a significant decrease in beta power with movement in both the GPi and STN, with higher beta power during rest and movement in the GPi, which also had more beta desynchronization during movement (30). Taken together, these results demonstrated that voluntary movement suppresses beta oscillations in the STN, a well-known phenomenon corresponding to movement-related desynchronization of beta routinely observed at the cortical level.

Moreover, a beta band suppression was reported in akinetic rigid patients during forward walking without detecting this behavior in tremor-dominant patients, suggesting that LFP modulation could also be related to PD phenotype (31).

These results underline the importance of beta-burst modulation in movement generation and impact the usefulness of LFP as a feedback signal for closed-loop stimulation, as the beta peaks suppression caused by movement could be erroneously interpreted as the absence of bradykinesia by the adaptive-DBS algorithm.

To note, in our study, the similarities between results, shown in Figure 2 and Supplementary Figure S2, suggest that, for the purpose of this study, Brainsense™ events recordings may provide enough information, and, hence, real-time streaming might not be strictly necessary.

In conclusion, subthalamic LFP in the beta band are considered as potential biomarkers for closed-loop DBS in PD. The beta band is further dissected into low-and high-frequency components, but their significance remains unclear and understood. This single exploratory case, on a PD patient with a double beta peak activity on both STN, illustrates a complex behavior of low-and high-beta peaks in response to stimulation, L-dopa plasma levels, and motor tasks. Our results suggest the importance of investigating patient-specific individual LFP patterns (LFP “fingerprints”), in order to optimize stimulation, to investigate predictive role of frequency behavior on patients’ outcomes, and also in view of upcoming closed-loop stimulation.

## Data availability statement

The raw data supporting the conclusions of this article will be made available by the authors, without undue reservation.

## Ethics statement

The studies involving human participants were reviewed and approved by the Ethics Committee of the Local Health Service of Bologna, Italy (Cod. CE18005-CE21156). Written informed consent to participate in this study was provided by the patient. Written informed consent was obtained from the individual for the publication of any potentially identifiable images or data included in this article.

## Author contributions

GG: conception and design of the study, acquisition, analysis and interpretation of data, and drafting of the manuscript. LB: acquisition, analysis and interpretation of data, and drafting of the manuscript. GLe: analysis and interpretation of data. IC and GLo: acquisition, and analysis and interpretation of data. PM: critical revision of the manuscript. PC and GC-B: substantial contributions to the conception and design of the study, and critical revision of the manuscript. AC: conception and design of the study, supervision of the study, and critical revision of the manuscript. All authors contributed to the article and approved the submitted version.

## Funding

The publication of this article was supported by the “Ricerca Corrente” funding from the Italian Ministry of Health.

## Conflict of interest

GL is an employee and shareholder of Medtronic PLC. Outside the present work, GC-B has received honoraria for speaking engagements or consulting activities from Abbvie, Bial, and Zambon.

The remaining authors declare that the research was conducted in the absence of any commercial or financial relationships that could be construed as a potential conflict of interest.

## Publisher’s note

All claims expressed in this article are solely those of the authors and do not necessarily represent those of their affiliated organizations, or those of the publisher, the editors and the reviewers. Any product that may be evaluated in this article, or claim that may be made by its manufacturer, is not guaranteed or endorsed by the publisher.

## Supplementary material

The Supplementary material for this article can be found online at: https://www.frontiersin.org/articles/10.3389/fneur.2023.1163811/full#supplementary-material
