# Supplementary figures and images for: Case report: Bilateral double beta peak activity is influenced by stimulation, levodopa concentrations, and motor tasks, in a Parkinson’s disease patient on chronic deep brain stimulation
Source: Front Neurol. 2023 May 18;14:1163811. doi: 10.3389/fneur.2023.1163811 (PMC10232856; doi:10.3389/fneur.2023.1163811)

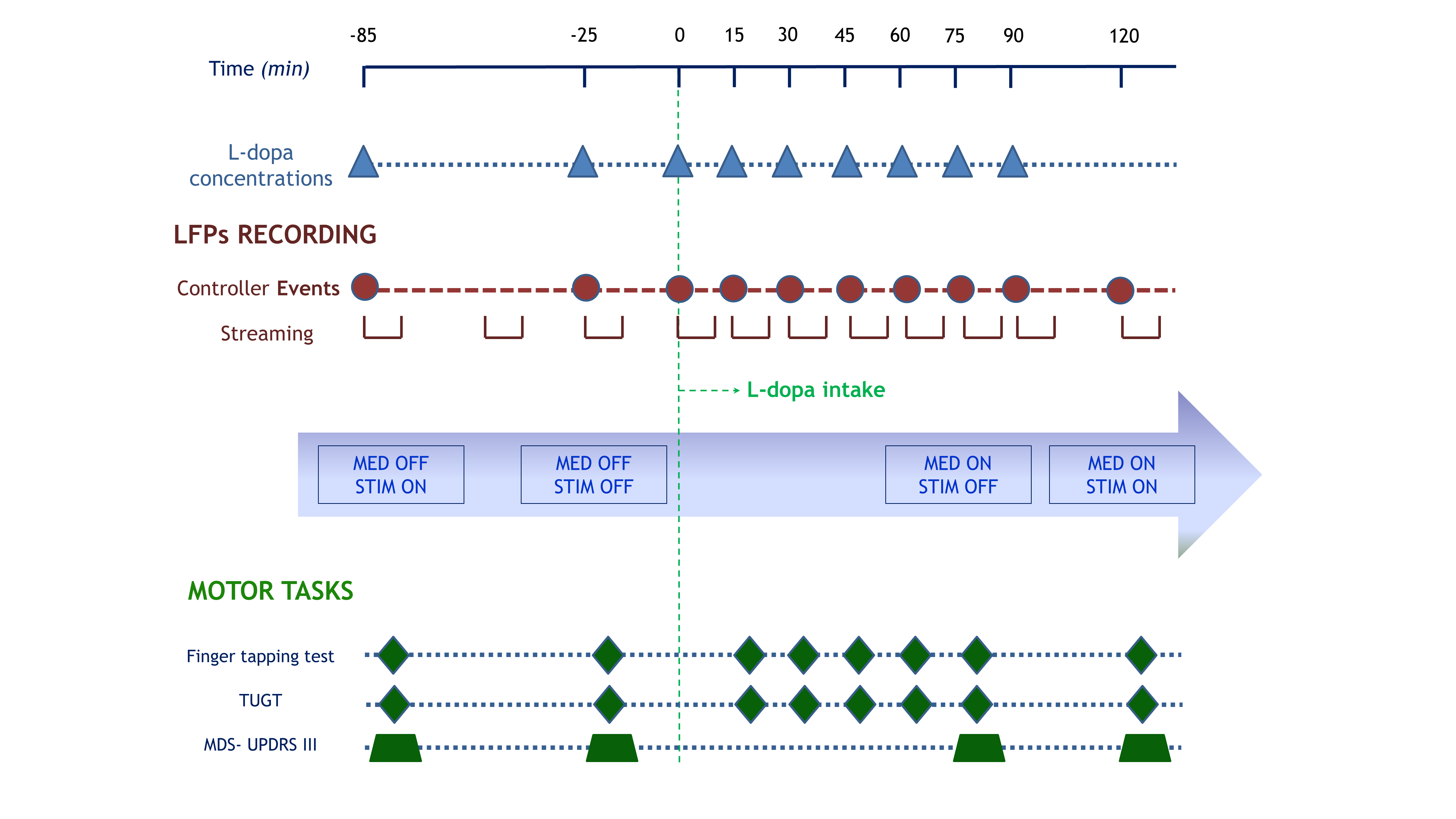

Supplement: Supplementary file 1 [file Image_1.TIF]
